# Supplementary material for: Integrative Application of Transcriptomics and Metabolomics Provides Insights into Unsynchronized Growth in Sea Cucumber (Stichopus monotuberculatus)
Source: Int J Mol Sci. 2022 Dec 7;23(24):15478. doi: 10.3390/ijms232415478 (PMC9779819; doi:10.3390/ijms232415478)
Supplement: Supplementary file 1 [file ijms-23-15478-s001.zip › Table S1.pdf]

Table S1. Comparison of the growth performance of *S. monotuberculatus* in the fast-growing and slow-growing groups.

**Group**	**Body Weight(g)**
SMF	3.3172±0.93000***
SMS	0.5483±0.08813

Note: “***” indicated the significant difference between growth performance in SMF and SMS groups (P<0.001).
